# Case report: A case of type 1 diabetes with diabetic ketoacidosis induced by envafolimab treatment in hepatocellular carcinoma

**DOI:** 10.3389/fimmu.2025.1505195

**Published:** 2025-01-28

**Authors:** Youjia Li, Kai Qu, Xiuli Li, Xin Yang, Kanghuai Zhang, Jiao Xie

**Affiliations:** ^1^ Department of Pharmacy, the Second Affiliated Hospital of Xi’an Jiaotong University, Xi’an, China; ^2^ Department of Hepatobiliary-Pancreatic and Liver Transplantation, The Second Affiliated Hospital of Xi’an Jiaotong University, Xi’an, China; ^3^ Department of Geriatric Endocrinology, the Second Affiliated Hospital of Xi′an Jiaotong University, Xi’an, China

**Keywords:** PD-L1, envafolimab, type 1 diabetes mellitus, diabetic ketoacidosis, immune checkpoint inhibitor, hepatocellular carcinoma

## Abstract

This case report presents a 57-year-old male with hepatocellular carcinoma who developed Type 1 Diabetes Mellitus (T1DM) and diabetic ketoacidosis (DKA) following treatment with Envafolimab, a PD-L1 immune checkpoint inhibitor. The patient experienced a rapid onset of hyperglycemia and DKA after several cycles of Envafolimab, consistent with the pattern of diabetes induced by immune checkpoint inhibitors (ICIs). Notably, the absence of diabetes-related autoantibodies suggests that the diabetes was induced by the immune-modulating effects of Envafolimab rather than a pre-existing autoimmune condition. Management required intensive insulin therapy and a multidisciplinary approach to stabilize the patient’s health. This case underscores the critical need for heightened clinical awareness and early intervention in managing severe immune-related adverse events (irAEs) associated with novel ICIs like Envafolimab. The complexity of autoimmune-related adverse events, such as the negative autoimmune profiles observed in our patient, emphasizes the importance of multidisciplinary collaboration to optimize patient outcomes. We advocate for the establishment of long-term follow-up plans, including regular monitoring for potential irAEs and endocrine function assessments, to address the chronicity of conditions post-ICI treatment. Recognizing the limitations of current understanding, there is a clear call for further research, particularly on identifying biomarkers that may predict adverse reactions to immunotherapy, to guide precision medicine and improve patient safety.

## Introduction

Malignant tumors are a leading cause of death worldwide, significantly affecting life expectancy. The GLOBOCAN 2020 statistics demonstrate that there were around 19.3 million new cancer cases and nearly 10 million cancer-related deaths globally, highlighting the substantial burden of this disease (1). In the face of such a significant global cancer burden, the search for effective treatment modalities has been relentless. Immunotherapy, particularly immune checkpoint inhibitors like programmed death-1 (PD-1) and its ligand (PD-L1), has emerged as a revolutionary approach in cancer treatment, demonstrating initial effectiveness across diverse malignancies (2). However, the response rates to immunotherapy are still relatively low, with only about 20% of patients experiencing benefits (3). This has led to an intensified search for immune predictive biomarkers that can help identify which populations are more likely to respond positively to immunotherapy.

Research has shown that tumors exhibiting microsatellite instability-high (MSI-H) or mismatch repair deficient (dMMR) characteristics tend to respond better to immunotherapy (4), making these biomarkers increasingly significant in clinical practice. Envafolimab, a humanized PD-L1 monoclonal antibody, is an immunotherapy drug that belongs to the category of immune checkpoint inhibitors (ICIs) and is a significant innovation in this field. It is the first PD-L1 inhibitor to be administered subcutaneously and also the first drug approved in China for broad-spectrum indications (MSI-H/dMMR) in the treatment of advanced solid tumors. It has received orphan drug designation from the U.S. FDA for advanced biliary tract cancer and soft tissue sarcoma, and in November 2021 (5), it was approved in China for patients with unresectable or metastatic adult solid tumors with MSI-H/dMMR who have experienced disease progression after treatment with fluorouracil, oxaliplatin, and irinotecan.

Immune-related adverse events (irAEs) defines various adverse reactions that occur during immunotherapy due to the activation of the immune system, resulting in autoimmune responses. Nevertheless, along with its promising therapeutic outcomes, the incidence of irAEs associated with Envafolimab is drawing increasing attention (6). The incidence of irAEs for Envafolimab is 24.0%. The most common irAEs (incidence ≥2%) include hypothyroidism, hyperthyroidism, immune-related hepatitis, and rash (7). These adverse effects encompass a range of issues, including dermatologic reactions, endocrine disorders, pulmonary complications, and hepatic dysfunction, all of which can significantly impair a patient’s quality of life. Of particular concern is the emerging link between Envafolimab and the onset of Type 1 Diabetes Mellitus (T1DM) (8). Although these complications are relatively rare, they can have severe implications, especially when accompanied by diabetic ketoacidosis (DKA), which poses considerable risks to patient health and can be life-threatening.

This case report details a patient with hepatocellular carcinoma who developed T1DM and DKA following treatment with Envafolimab. By documenting this case, we aim to raise clinical awareness about the adverse effects associated with Envafolimab, underscoring the necessity for early detection and timely management. This report represents the first documented instance of T1DM triggered by Envafolimab, highlighting the critical need for ongoing research and refined treatment strategies within the realm of immunotherapy.

## Case presentation

In April 2021, a 57-year-old male patient, with a height of 165 cm and a weight of 67 kg, had a Body Mass Index (BMI) of 24.61 kg/m² (which is above the normal range of 18.5 to 23.9 kg/m²) and was admitted for surgical intervention due to a lumbar vertebral fracture sustained from a traumatic event. During the surgical procedure, he tested positive for hepatitis B markers, specifically for the hepatitis B surface antigen, and had elevated quantitative hepatitis B surface antigen levels, though the exact values were not provided. An abdominal ultrasound revealed liver cirrhosis, leading to the initiation of oral antiviral treatment with entecavir. In October 2022, during routine follow-up, the patient presented with elevated abnormal prothrombin levels (PIVKA-II). Due to the patient’s treatment at an external facility, the specific values were not recorded and are currently unobtainable. A contrast-enhanced ultrasound indicated the presence of regenerative nodules in the liver. This prompted a recommendation for further magnetic resonance imaging using the hepatobiliary-specific contrast agent Gd-EOB-DTPA (Primovist) to rule out malignant liver tumors; however, the patient declined this additional examination due to financial constraints. By April 2023, his abnormal prothrombin level had escalated to 951.94 mAU/mL, significantly above the normal range of 15.40-43.56 mAU/mL. A subsequent contrast ultrasound indicated multiple high-echo lesions in the liver, which were tentatively diagnosed as regenerative nodules, and he continued his antiviral treatment with entecavir.

Two months before his admission, the patient experienced symptoms including abdominal distension, fatigue, decreased appetite, yellowing of urine, and occasional nausea. These symptoms prompted his admission to the Department of Hepatobiliary, Pancreatic, and Transplant Surgery at Xi’an Jiaotong University Second Affiliated Hospital on June 12, 2023. The patient has a smoking history spanning over 20 years, during which he smoked approximately 15 cigarettes daily, and he consumed a small amount of alcohol. The impact of smoking and alcohol on liver disease progression is significant; both are known risk factors that can exacerbate liver conditions and may affect response to antiviral treatment. However, he has been abstinent from both for the past two years. He reported no history of chronic diseases such as hypertension, coronary artery disease, or diabetes, and he also denied any previous infectious diseases, significant surgical procedures, blood transfusions, or allergies to drugs or food. Furthermore, he did not report any exposure to epidemic areas, contaminated water, hazardous chemicals, or radioactive materials.

**Figure 1:**
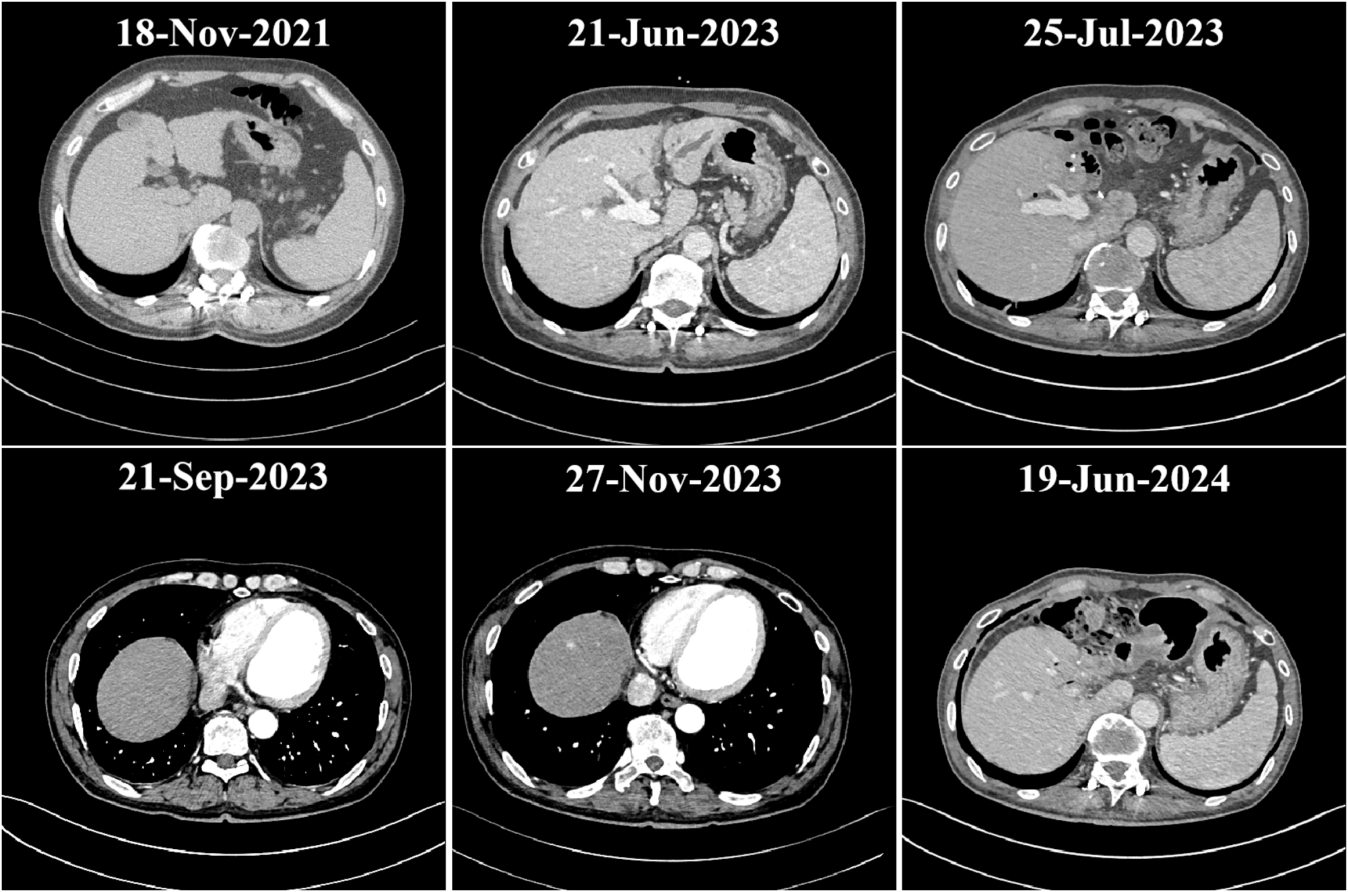
Image of disease progression.

**Figure 2:**
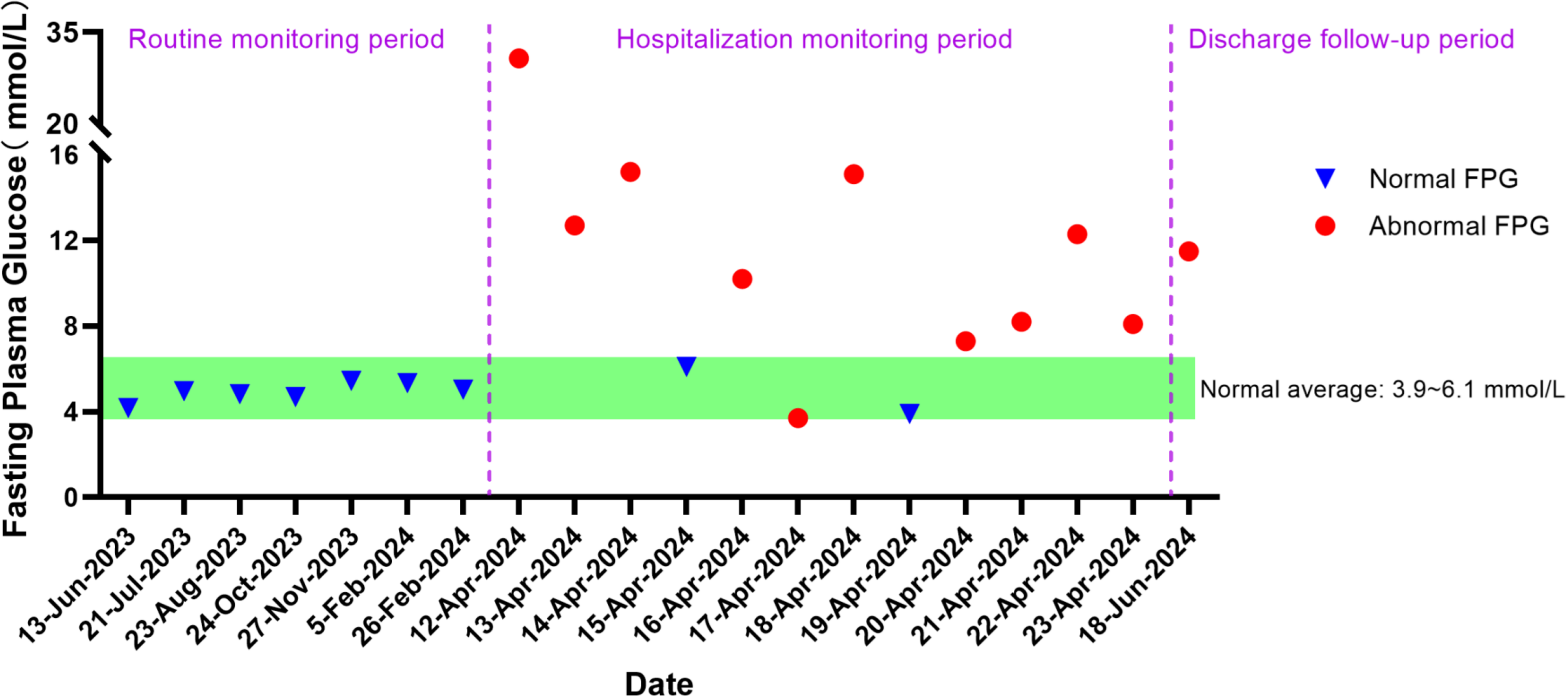
Fasting Plasma Glucose monitoring results of the patient at different time points.

Upon admission, further examinations confirmed a diagnosis of hepatocellular carcinoma, which was specifically moderately differentiated (Stage IVA, T3N1M0). On June 27, 2023, the patient underwent radical surgery for cholangiocarcinoma, which involved liver resection due to porta hepatis ossification, bile duct resection, left liver lobe resection, and biliary-enteric anastomosis, and exhibited satisfactory postoperative recovery. From July 23, 2023, he commenced targeted therapy with 8 mg of lenvatinib capsules twice daily, followed by immunotherapy with an injection of Envafolimab (400 mg, subcutaneous injection). Envafolimab was administered over 10 cycles, on specific dates: August 24, September 20, October 24, December 23, January 15, February 5 and 26, March 15, and April 9 of 2024. Each dose of Envafolimab was 400 mg, administered via subcutaneous injection. A follow-up CT on November 27, 2023, indicated a newly developed abnormal enhancing nodule at the liver apex compared to the scan on September 20 (**Figure 1**). Subsequently, on December 22, 2023, he underwent transarterial chemoembolization (TACE), receiving treatment with “Oxaliplatin 50 mg, Levoleucovorin 100 mg, and Fluorouracil 250 mg.” By February 26, 2024, the patient’s fasting blood glucose and glycated albumin levels remained within normal reference ranges (**Figure 2**).

**Figure 3:**
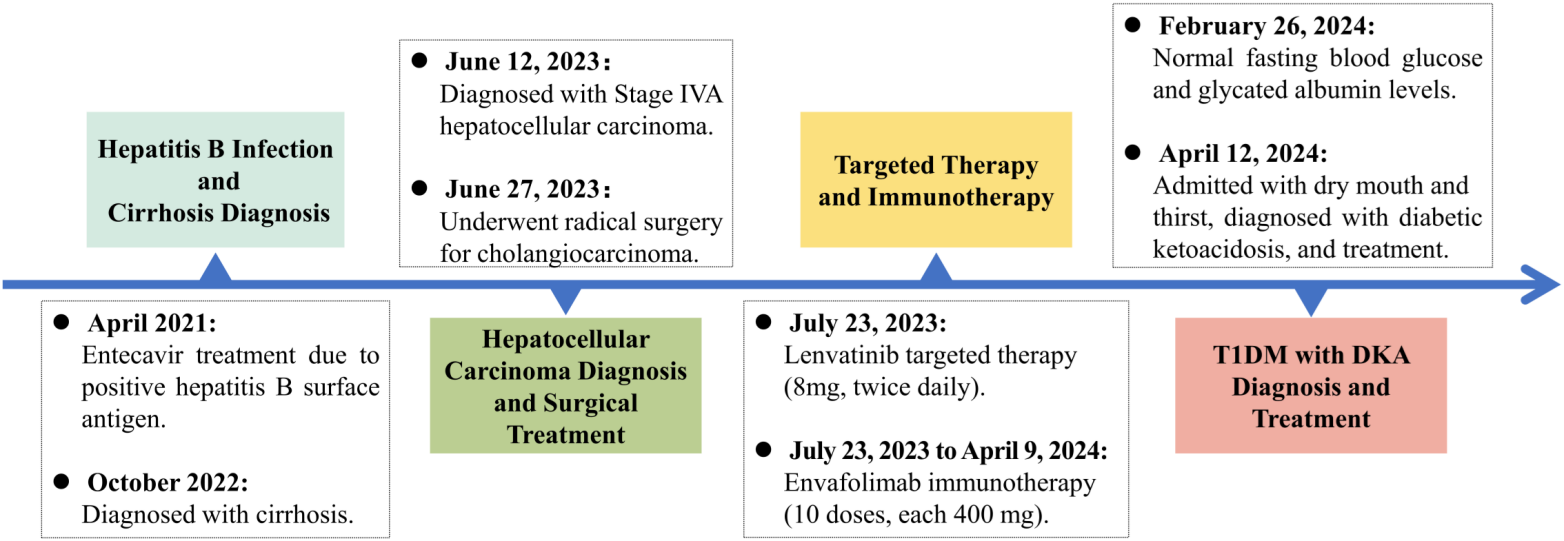
Timeline of disease progression. T1DM, Type 1 Diabetes Mellitus; DKA, Diabetic Ketoacidosis.

On April 12, 2024, the patient presented with dry mouth and increased thirst, alongside fatigue, prompting his readmission to the Department of Hepatobiliary, Pancreatic, and Transplant Surgery. Physical examination revealed a height of 165 cm and weight of 58 kg (approximately a 9 kg weight loss over the past month), with a BMI of 21.30 kg/m². Vital signs included T: 36.5°C, P: 67 beats/min, R: 16 breaths/min, and a blood pressure: 119/82 mmHg. Admission laboratory tests revealed random fingertip blood glucose levels of 29.3 mmol/L and random venous blood glucose levels of 30.73 mmol/L (**Figure 2**), along with a glycated albumin at 28.25% and HbA_1c_ of 8.74%. Urinalysis showed a urine glucose level of 4+ and a urine ketones level of 3+. Arterial blood gas analysis indicated a blood pH of 7.227 and a pCO2 of 27.6 mmHg. He was diagnosed with diabetic ketoacidosis and received a continuous intravenous insulin infusion at a rate of 4-5 U/h, with blood glucose levels monitored every 1-2 hours and adjustments made to the insulin administration rate and dosage accordingly. Supportive treatments including rehydration and correction of ketoacidosis were also initiated. After correction of acidosis, the patient was transferred to the endocrinology department for continued glycemic management, receiving continuous subcutaneous insulin infusions (aspart insulin) with a basal dose of 15.6 U/day and prandial doses of 5 U before each meal, with blood glucose levels monitored for insulin dose adjustments. Once glycemic control was stabilized, fasting C-peptide levels were measured at 0.04 ng/ml, and postprandial 2-hour C-peptide levels at 0.03 ng/ml; all five insulin autoantibody tests were negative. Considering the patient’s clinical presentation and laboratory results, such as C-peptide, HbA_1c_, ketones, and pH values, he was diagnosed with type 1 diabetes combined with diabetic ketoacidosis, which was thought to be induced by the immune checkpoint inhibitor, Envafolimab. Main diagnostic and endocrine-related indicators during hospitalization are summarized in **Table 1**. **Figure 3** illustrates the timeline of Envafolimab treatment and the onset of T1DM with DKA.

**Table 1:** Main diagnostic indicators and endocrine-related indicators during patient hospitalization.

Category	Indicator Item	Monitoring Result	Reference Range	Unit
Arterial Blood Gas Analysis	Arterial Blood Gas pH Value	7.227	7.350-7.450	
	Arterial Blood Gas pCO2	27.6	35.0-45.0	mmHg
Urine Analysis	Urine Ketones	3+	Negative	
	Urine Glucose	4+	Negative	
Glycemic Control Indicators	HbA_1c_	8.74	4-6	%
	Glycated Albumin	28.25	10.8-17.1	%
	Fasting C-peptide	0.04	0.4-5.7	ng/ml
Insulin Secretion Function	Postprandial 2h C-peptide	0.03		ng/ml
	Fasting Insulin	9.80	1.9-23	uU/ml
	Postprandial 2h Insulin	27.20		uU/ml
Autoimmune Diabetes Antibodies	Insulin Autoantibodies (IAA)	0.25	<1.0	COI
	Glutamic Acid Decarboxylase Antibodies (GADA)	0.81	<10	IU/ml
	Insulinoma-Associated Protein Antibodies (IA-2A)	1.28	<10	IU/ml
	Islet Cell Autoantibodies (ICA)	0.17	<1.0	COI
	Zinc Transporter 8 Antibodies (ZnT8)	<1.00	<10	AU/ml

Following successful glycemic control, the patient was discharged with a regimen of 12 U of degludec insulin for subcutaneous injection at bedtime, combined with aspart insulin (5 U, 5 U, 2 U) before each meal. At a two-month follow-up in the outpatient setting, fasting blood glucose levels were recorded at 11.49 mmol/L (**Figure 2**), with postprandial 2-hour glucose fluctuations between 8.5 and 20.3 mmol/L and glycated albumin at 29.81%. Repeat fasting C-peptide levels were <0.01 ng/ml, and postprandial 2-hour C-peptide levels were also <0.01 ng/ml, prompting further adjustments to insulin dosages. The pharmacist provided medication education, and the patient continues to be monitored during specialized outpatient follow-ups.

## Discussion

The case of a 57-year-old male with hepatocellular carcinoma who developed Type 1 Diabetes Mellitus (T1DM) and diabetic ketoacidosis (DKA) following treatment with the immune checkpoint inhibitor Envafolimab is significant for several reasons. Type 1 diabetes related to immune checkpoint inhibitors is a rare yet serious adverse event associated with their use. This case underscores the potential for severe irAEs associated with ICIs, highlighting the necessity for vigilant monitoring and prompt management of these complications.

The burgeoning field of immunotherapy, particularly the use of ICIs like Envafolimab, has revolutionized cancer treatment by leveraging the immune system to target and destroy cancer cells (9). While these therapies have shown substantial efficacy in treating a variety of malignancies, they are not without risks. Immune-related adverse events, including endocrinopathies such as T1DM, are increasingly recognized as significant complications of ICI therapy (10). The incidence of ICI-induced diabetes, although relatively rare, is a serious concern due to its acute onset and potential for life-threatening conditions like DKA (11).

Like other immune checkpoint inhibitors, Envafolimab is associated with irAEs that can affect multiple organ systems (12). In a phase II study involving 103 patients with previously treated advanced dMMR/MSI-H solid tumors, Envafolimab demonstrated a favorable safety profile with manageable irAEs (13). The most common irAEs included hypothyroidism, hyperthyroidism, and immune-associated hepatitis, with grade 3 or 4 events occurring in 8% of patients. These studies indicate that while Envafolimab is generally well-tolerated, the potential for severe irAEs necessitates close monitoring.

A prospective, open-label, single-arm phase II clinical trial was conducted to assess the safety and efficacy of the combination therapy of envafolimab and lenvatinib alongside transarterial chemoembolization in patients with unresectable hepatocellular carcinoma, involving a cohort of 37 participants (14). The overall frequency of treatment-related adverse events (TRAEs) of any severity was recorded at 97.4% (36/37) of patients. Notably, TRAEs classified as grade 3 or higher were noted in 52.6% (20/37) of patients, with no reported cases of hyperglycemia or diabetes linked to the treatment. In a separate retrospective study examining the effects of Envafolimab in a sample of 64 patients with advanced malignant solid tumors, adverse events were identified in 68.8% (44/64) of patients, with severe adverse events (CTCAE grade 3/4) occurring in 14.1% (9/64)of patients. The majority of these adverse events were of mild severity, resulting in treatment cessation for only 3.1% (44/64) of patients, and no life-threatening incidents were documented (15).Importantly, adverse events classified as “hyperglycemia” occurred in 15 patients (24.3%), although a definitive diagnosis of diabetes was not established.

To assess the prevalence of immune checkpoint inhibitor-related diabetes mellitus (ICI-DM), a review of phase 3 clinical trials involving immune checkpoint inhibitors (ICIs) and published case reports concerning ICI-DM was conducted by other researchers (16), indicating that the incidence of ICI-DM is below 1%. This assessment included a retrospective analysis of agents such as Nivolumab, Pembrolizumab, Atezolizumab, and Durvalumab; however, Envafolimab was notably absent from this analysis, likely due to a scarcity of reports associating Envafolimab with diabetes. Among the 109 documented cases of ICI-DM, the majority of patients were male, with a mean age of 62 years. The onset of ICI-DM was observed as early as one week and as late as 26 months post-initial ICI treatment, with a median onset time of 13 weeks (ranging from 6.0 to 26.3 weeks) (16). This timeline is consistent with the occurrence of diabetes attributed to Envafolimab as reported in this study.

Current literature indicates that the pathogenesis of ICI-induced T1DM involves immune-mediated destruction of pancreatic β-cells, similar to the autoimmune process observed in spontaneous T1DM. Specifically, PD-L1 inhibition can lead to the activation of CD4+ and CD8+ T cells, which release pro-inflammatory cytokines such as interferon-γ and tumor necrosis factor-α (17). This cytokine surge contributes to the recruitment of additional immune cells and induces apoptosis in β-cells. Furthermore, the loss of PD-L1 signaling disrupts the immune tolerance normally maintained against β-cells, amplifying the autoimmune response (18). However, the precise mechanisms remain to be fully elucidated in this context. Studies have indicated that in certain cases, patients may not exhibit traditional autoimmune markers such as GAD65 or IA-2 autoantibodies, which may suggest an alternate mechanism of immune response induced by ICI therapy (19). The absence of these markers does not preclude the diagnosis of Type 1 Diabetes, as some patients may present with a unique immunological profile following ICI treatment.

Moreover, research has documented instances of ICI-induced diabetes exhibiting negative autoimmune profiles, highlighting that the immune-mediated destruction of β-cells can occur without the presence of conventional autoimmune markers (20). Studies have suggested that ICIs, by blocking inhibitory pathways such as PD-1/PD-L1, may inadvertently enhance T-cell activity against pancreatic β-cells, leading to their destruction and subsequent insulin deficiency (21). This phenomenon has been observed with various ICIs, including pembrolizumab, nivolumab, and ipilimumab, with reported cases of rapid-onset diabetes and severe insulin deficiency (22). When comparing this case with other documented cases of ICI-induced T1DM in the literature, our patient’s presentation shares similarities with those associated with other PD-1/PD-L1 inhibitors, such as rapid-onset hyperglycemia and DKA following treatment initiation. However, the absence of diabetes-related autoantibodies in our patient, which is not commonly reported, suggests a potentially unique aspect of Envafolimab-induced T1DM. This finding emphasizes the need for further research to understand the distinct mechanisms and clinical presentations of T1DM associated with different ICIs.

In the context of Envafolimab, the first subcutaneously administered PD-L1 inhibitor, this case report is notably the first to document T1DM as an adverse event. The patient’s clinical course, characterized by the abrupt onset of hyperglycemia and DKA following multiple cycles of Envafolimab, aligns with the pattern of ICI-induced diabetes described in other reports (23). The absence of diabetes-related autoantibodies in this patient further supports the hypothesis that the diabetes was induced by the immune-modulating effects of Envafolimab rather than a pre-existing autoimmune condition (24).

When managing ICI-induced T1DM, a multidisciplinary approach is essential, involving endocrinologists and oncologists to ensure optimal glycemic control and continuous monitoring for potential long-term complications. The onset of ICI-DM is challenging to predict. Patients undergoing ICI therapy should be informed about hyperglycemia signs and routinely monitor blood glucose levels during each chemotherapy cycle (25). In this case, the patient required intensive insulin therapy and close monitoring to stabilize blood glucose levels and manage DKA, followed by a maintenance regimen of basal and prandial insulin. This approach is consistent with the recommendations for managing ICI-induced endocrinopathies, emphasizing early detection and aggressive treatment to mitigate morbidity and mortality (26). In terms of alternative management strategies, various insulin regimens have been considered, including basal-bolus and premixed insulin therapies, with the selection often tailored to the patient’s specific needs and response to treatment. Additionally, the role of continuous glucose monitoring systems (CGM) in the management of ICI-induced T1DM has been explored, providing real-time glucose data to guide insulin dosing and minimize hypoglycemic events. The integration of these strategies into a comprehensive care plan is crucial for achieving optimal outcomes in patients with ICI-induced T1DM.

In conclusion, this case underscores the critical need for heightened clinical awareness and early intervention in managing severe irAEs, including T1DM, associated with novel ICIs like Envafolimab. The complexity of autoimmune-related adverse events, such as the negative autoimmune profiles observed in our patient, emphasizes the importance of multidisciplinary collaboration to optimize patient outcomes. We advocate for the establishment of long-term follow-up plans, including regular monitoring for potential irAEs and endocrine function assessment, to address the chronicity of conditions post-ICI treatment. Recognizing the limitations of current understanding, there is a clear call for further research, particularly on identifying biomarkers that may predict adverse reactions to immunotherapy, to guide precision medicine and improve patient safety.

## Data Availability

The original contributions presented in the study are included in the article/supplementary material, further inquiries can be directed to the corresponding author/s.
